# Second monoclinic modification of cyclo­hexane-1,1-dicarbonitrile

**DOI:** 10.1107/S1600536811023592

**Published:** 2011-06-25

**Authors:** Nurlana D. Sadikhova, Ali. N. Khalilov, Atash V. Gurbanov, Iván Brito

**Affiliations:** a Department of Organic Chemistry, Baku State University, Baku, Azerbaijan; b Departamento de Química, Facultad de Ciencias Básicas, Universidad de Antofagasta, Casilla 170, Antofagasta, Chile

## Abstract

In the title compound, C_8_H_10_N_2_, the cyclo­hexane ring adopts a chair conformation. he crystal structure of the previously reported monoclinic modification have intramolecular CN⋯CN and C—H⋯N interactions. These types of interaction are not present in this new modification whose crystal structure is built up by van der Waals interactions.

## Related literature

For the previously reported monoclinic modification, see: Echeverria *et al.* (1995 ▶). For synthetic methods, see: Tsai *et al.* (2003 ▶); Suissa *et al.* (1977 ▶); Julia & Maumy (1969 ▶). For puckering parameters see: Cremer & Pople (1975 ▶).
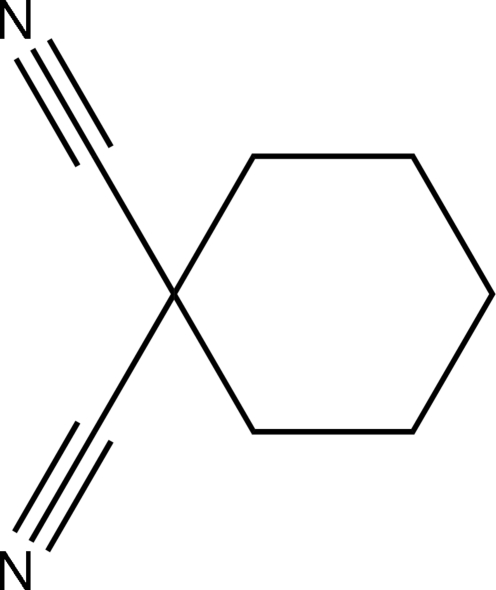


## Experimental

### 

#### Crystal data

C_8_H_10_N_2_
*M* *_r_* = 134.18
Monoclinic, 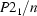

*a* = 8.9300 (5) Å
*b* = 8.3656 (5) Å
*c* = 9.8725 (6) Å
β = 92.662 (1)°
*V* = 736.73 (8) Å^3^
*Z* = 4
Mo *K*α radiation
μ = 0.08 mm^−1^
*T* = 100 K
0.30 × 0.30 × 0.30 mm

#### Data collection

Bruker APEXII CCD diffractometer
Absorption correction: multi-scan (*SADABS*; Sheldrick, 2001 ▶) *T*_min_ = 0.978, *T*_max_ = 0.978
9584 measured reflections
2794 independent reflections
2236 reflections with *I* > 2σ(*I*)
*R*_int_ = 0.033

#### Refinement

*R*[*F* ^2^ > 2σ(*F* ^2^)] = 0.040
*wR*(*F* ^2^) = 0.105
*S* = 1.03
2794 reflections
91 parameters
H-atom parameters constrained
Δρ_max_ = 0.31 e Å^−3^
Δρ_min_ = −0.21 e Å^−3^

### 

Data collection: *APEX2* (Bruker, 2005 ▶); cell refinement: *SAINT* (Bruker, 2005 ▶); data reduction: *SAINT*; program(s) used to solve structure: *SHELXS97* (Sheldrick, 2008 ▶); program(s) used to refine structure: *SHELXL97* (Sheldrick, 2008 ▶); molecular graphics: *ORTEP-3 for Windows* (Farrugia, 1997 ▶); software used to prepare material for publication: *WinGX* (Farrugia, 1999 ▶).

## Supplementary Material

Crystal structure: contains datablock(s) I, global. DOI: 10.1107/S1600536811023592/ng5170sup1.cif

Structure factors: contains datablock(s) I. DOI: 10.1107/S1600536811023592/ng5170Isup2.hkl

Supplementary material file. DOI: 10.1107/S1600536811023592/ng5170Isup3.cml

Additional supplementary materials:  crystallographic information; 3D view; checkCIF report

## Figures and Tables

**Fig. 1.**
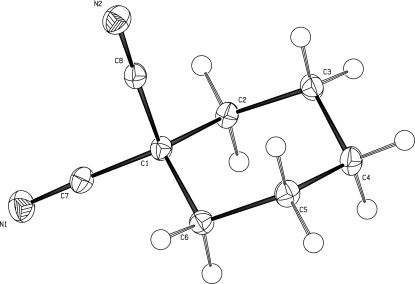
The structure of (I), showing the atom-numbering scheme. Displacement ellipsoids are plotted at the 30% probability level.

## supplementary crystallographic information

### Comment

Fig.1 shows the structure of title compound, (I) which is a polymorphic form of a previously reported by (Echeverria *et al.*, 1995), (II). The bond distances and the six C—C—C bond angles in (I) are slightly longer than (II). The crystal structure of (II) have intermolecular CN···CN and C—H···N interactions, whereas this kind of interactions are not present in (I). The values of the ring puckering parameters: Q_T_ = 0.5665 Å, θ =0.72° and φ=107.4° (Cremer & Pople, 1975), indicate that the cyclohexane has a chair conformation. The C1—C2 and C1—C6 bond distances are more longer than the other C—C distances in the cyclohexyl ring. The lengthening of these bonds with increasing ring size may be attributed to steric crowding about C1 atom. The cyano groups are essentially collinear with C1 and the N—C—C1 angles is 178.55 (10)° (mean). A σ_h_ plane passing through the CN groups and the C1 atom which bisects the cyclohexyl ring.

### Experimental

A mixture of malonodinitrile (0.1 mol, 6.6 g), 23 gr 1,5-dibromopentane (0.1 mol, 23 g) and 46 gr K_2_CO_3_ in dry DMSO (50 ml), was stirred for 12 h at 70 °C. After cooling down, the reaction mixture was poured into water and extracted with ether. The organic layer was washed several times with water, dried over Na_2_SO_4_ and the solvent evaporated. The crude product was purified by vacuum distillation yielding 10.5 gr (87%) of a solid compound which after recrystallization in hexane gave white crystals: mp 65 °C.; ^1^H NMR (300 MHz, CDCI_3_) δ 1.52 (m, 2H, CH_2_), 1.72 (m, 4H, 2CH_2_), 2.12 (t, 4H,2CH_2_); ^13^CNMR (75 MHz, CDCI_3_) δ 22.3, 23.8, 34.7, 41.2, 117.3. Analysis, found, %: C: 71.42, H: 7.78, N: 20.63 (C_8_H_10_N_2_); calculated, %: C: 71.61, H: 7.51, N: 20.88

### Refinement

One reflection (-7 1 3) was omitted of the refinement due to to bad agreement between observed and calculated factors. All H-atoms were placed in calculated positions [C—H = 0.99 Å, *U*_iso_(H) = 1.2 *U*_eq_(C)] and were included in the refinement in the riding model approximation.

### Crystal data

**Table:**

C_8_H_10_N_2_	*F*(000) = 288
*M**_r_* = 134.18	*D*_x_ = 1.210 Mg m^−^^3^
Monoclinic, *P*2_1_/*n*	Mo *K*α radiation, λ = 0.71073 Å
Hall symbol: -P 2yn	Cell parameters from 2571 reflections
*a* = 8.9300 (5) Å	θ = 3.0–33.1°
*b* = 8.3656 (5) Å	µ = 0.08 mm^−^^1^
*c* = 9.8725 (6) Å	*T* = 100 K
β = 92.662 (1)°	Prism, colourless
*V* = 736.73 (8) Å^3^	0.30 × 0.30 × 0.30 mm
*Z* = 4	

### Data collection

**Table:**

Bruker APEXII CCD diffractometer	2794 independent reflections
Radiation source: fine-focus sealed tube	2236 reflections with *I* > 2σ(*I*)
graphite	*R*_int_ = 0.033
φ and ω scans	θ_max_ = 33.2°, θ_min_ = 3.0°
Absorption correction: multi-scan (*SADABS*; Sheldrick, 2001)	*h* = −13→13
*T*_min_ = 0.978, *T*_max_ = 0.978	*k* = −12→12
9584 measured reflections	*l* = −15→14

### Refinement

**Table:**

Refinement on *F*^2^	Primary atom site location: structure-invariant direct methods
Least-squares matrix: full	Secondary atom site location: difference Fourier map
*R*[*F*^2^ > 2σ(*F*^2^)] = 0.040	Hydrogen site location: difference Fourier map
*wR*(*F*^2^) = 0.105	H-atom parameters constrained
*S* = 1.03	*w* = 1/[σ^2^(*F*_o_^2^) + (0.0492*P*)^2^ + 0.1357*P*] where *P* = (*F*_o_^2^ + 2*F*_c_^2^)/3
2794 reflections	(Δ/σ)_max_ < 0.001
91 parameters	Δρ_max_ = 0.31 e Å^−^^3^
0 restraints	Δρ_min_ = −0.21 e Å^−^^3^

### Special details

**Table:**

Geometry. All s.u.'s (except the s.u. in the dihedral angle between two l.s. planes) are estimated using the full covariance matrix. The cell s.u.'s are taken into account individually in the estimation of s.u.'s in distances, angles and torsion angles; correlations between s.u.'s in cell parameters are only used when they are defined by crystal symmetry. An approximate (isotropic) treatment of cell s.u.'s is used for estimating s.u.'s involving l.s. planes.
Refinement. Refinement of *F*^2^ against ALL reflections. The weighted *R*-factor *wR* and goodness of fit *S* are based on *F*^2^, conventional *R*-factors *R* are based on *F*, with *F* set to zero for negative *F*^2^. The threshold expression of *F*^2^ > 2σ(*F*^2^) is used only for calculating *R*-factors(gt) *etc*. and is not relevant to the choice of reflections for refinement. *R*-factors based on *F*^2^ are statistically about twice as large as those based on *F*, and *R*- factors based on ALL data will be even larger.

### Fractional atomic coordinates and isotropic or equivalent isotropic displacement parameters (Å^2^)

**Table:**

	*x*	*y*	*z*	*U*_iso_*/*U*_eq_	
N1	0.36573 (9)	−0.12113 (9)	0.57950 (8)	0.02114 (17)	
N2	−0.02890 (8)	0.15605 (9)	0.63201 (8)	0.01948 (16)	
C1	0.24232 (8)	0.16105 (9)	0.53895 (8)	0.01226 (14)	
C2	0.23203 (9)	0.19844 (9)	0.38467 (8)	0.01370 (15)	
H2A	0.3327	0.1888	0.3476	0.016*	
H2B	0.1652	0.1197	0.3376	0.016*	
C3	0.17144 (9)	0.36675 (10)	0.35831 (9)	0.01563 (16)	
H3A	0.0672	0.3734	0.3878	0.019*	
H3B	0.1701	0.3895	0.2598	0.019*	
C4	0.26762 (9)	0.49156 (9)	0.43440 (9)	0.01663 (16)	
H4A	0.2237	0.5990	0.4185	0.020*	
H4B	0.3697	0.4915	0.3992	0.020*	
C5	0.27741 (9)	0.45676 (10)	0.58644 (8)	0.01565 (16)	
H5A	0.3437	0.5367	0.6326	0.019*	
H5B	0.1765	0.4671	0.6230	0.019*	
C6	0.33813 (9)	0.28925 (9)	0.61680 (8)	0.01401 (15)	
H6A	0.3367	0.2685	0.7155	0.017*	
H6B	0.4433	0.2823	0.5898	0.017*	
C7	0.30994 (9)	0.00103 (10)	0.56201 (8)	0.01494 (15)	
C8	0.08948 (9)	0.15673 (9)	0.59186 (8)	0.01406 (15)	

### Atomic displacement parameters (Å^2^)

**Table:**

	*U*^11^	*U*^22^	*U*^33^	*U*^12^	*U*^13^	*U*^23^
N1	0.0225 (3)	0.0166 (3)	0.0245 (4)	0.0026 (3)	0.0035 (3)	0.0023 (3)
N2	0.0181 (3)	0.0193 (3)	0.0213 (4)	−0.0012 (3)	0.0039 (3)	−0.0003 (3)
C1	0.0127 (3)	0.0109 (3)	0.0134 (3)	0.0006 (2)	0.0021 (2)	0.0006 (2)
C2	0.0170 (3)	0.0128 (3)	0.0115 (3)	−0.0012 (3)	0.0017 (3)	−0.0003 (3)
C3	0.0179 (3)	0.0144 (3)	0.0145 (3)	0.0001 (3)	−0.0010 (3)	0.0019 (3)
C4	0.0214 (4)	0.0116 (3)	0.0169 (4)	−0.0007 (3)	0.0006 (3)	0.0011 (3)
C5	0.0188 (3)	0.0124 (3)	0.0157 (4)	0.0003 (3)	0.0003 (3)	−0.0021 (3)
C6	0.0141 (3)	0.0136 (3)	0.0141 (3)	−0.0002 (2)	−0.0007 (3)	−0.0009 (3)
C7	0.0156 (3)	0.0143 (3)	0.0152 (4)	−0.0005 (3)	0.0032 (3)	0.0007 (3)
C8	0.0161 (3)	0.0125 (3)	0.0136 (3)	−0.0002 (3)	0.0009 (3)	0.0004 (3)

### Geometric parameters (Å, °)

**Table:**

N1—C7	1.1465 (11)	C3—H3A	0.9900
N2—C8	1.1462 (11)	C3—H3B	0.9900
C1—C7	1.4818 (11)	C4—C5	1.5275 (12)
C1—C8	1.4843 (11)	C4—H4A	0.9900
C1—C6	1.5530 (11)	C4—H4B	0.9900
C1—C2	1.5535 (11)	C5—C6	1.5272 (11)
C2—C3	1.5265 (11)	C5—H5A	0.9900
C2—H2A	0.9900	C5—H5B	0.9900
C2—H2B	0.9900	C6—H6A	0.9900
C3—C4	1.5272 (11)	C6—H6B	0.9900
			
C7—C1—C8	107.39 (6)	C3—C4—H4A	109.4
C7—C1—C6	109.67 (6)	C5—C4—H4A	109.4
C8—C1—C6	109.70 (6)	C3—C4—H4B	109.4
C7—C1—C2	109.73 (6)	C5—C4—H4B	109.4
C8—C1—C2	109.66 (6)	H4A—C4—H4B	108.0
C6—C1—C2	110.63 (6)	C6—C5—C4	111.80 (7)
C3—C2—C1	110.93 (6)	C6—C5—H5A	109.3
C3—C2—H2A	109.5	C4—C5—H5A	109.3
C1—C2—H2A	109.5	C6—C5—H5B	109.3
C3—C2—H2B	109.5	C4—C5—H5B	109.3
C1—C2—H2B	109.5	H5A—C5—H5B	107.9
H2A—C2—H2B	108.0	C5—C6—C1	110.74 (6)
C2—C3—C4	111.10 (6)	C5—C6—H6A	109.5
C2—C3—H3A	109.4	C1—C6—H6A	109.5
C4—C3—H3A	109.4	C5—C6—H6B	109.5
C2—C3—H3B	109.4	C1—C6—H6B	109.5
C4—C3—H3B	109.4	H6A—C6—H6B	108.1
H3A—C3—H3B	108.0	N1—C7—C1	178.29 (8)
C3—C4—C5	110.99 (7)	N2—C8—C1	178.83 (8)
			
C7—C1—C2—C3	−176.59 (6)	C8—C1—C6—C5	−66.44 (8)
C8—C1—C2—C3	65.69 (8)	C2—C1—C6—C5	54.66 (8)
C6—C1—C2—C3	−55.45 (8)	C8—C1—C7—N1	−161 (3)
C1—C2—C3—C4	56.61 (9)	C6—C1—C7—N1	−42 (3)
C2—C3—C4—C5	−56.87 (9)	C2—C1—C7—N1	80 (3)
C3—C4—C5—C6	56.58 (9)	C7—C1—C8—N2	−179 (100)
C4—C5—C6—C1	−55.55 (9)	C6—C1—C8—N2	62 (4)
C7—C1—C6—C5	175.84 (7)	C2—C1—C8—N2	−60 (4)
